# Scanning for satisfaction or digging for dismay? Comparing findings from a postal survey with those from a focus group-study

**DOI:** 10.1186/1471-2288-12-134

**Published:** 2012-09-03

**Authors:** Benedicte Carlsen, Claire Glenton

**Affiliations:** 1 Uni Rokkan Centre, Nygaardsgt 5, N-5015 , Bergen, Norway; 2 The Norwegian Knowledge Centre for the Health Services, PO Box 7004, St. Olavs plass, N-0130, Oslo, Norway

**Keywords:** Research methodology, Focus groups, Qualitative, Surveys, Mixed methods, Primary care physicians, GPs

## Abstract

**Background:**

Despite growing support for mixed methods approaches we still have little systematic knowledge about the consequences of combining surveys and focus groups. While the methodological aspects of questionnaire surveys have been researched extensively, the characteristics of focus group methodology are understudied. We suggest and discuss whether the focus group setting, as compared to questionnaire surveys, encourages participants to exaggerate views in a negative direction.

**Discussion:**

Based on an example from our own research, where we conducted a survey as a follow up of a focus group study, and with reference to theoretical approaches and empirical evidence from the literature concerning survey respondent behaviour and small group dynamics, we discuss the possibility that a discrepancy in findings between the focus groups and the questionnaire reflects characteristics of the two different research methods. In contrast to the survey, the focus group study indicated that doctors were generally negative to clinical guidelines. We were not convinced that this difference in results was due to methodological flaws in either of the studies, and discuss instead how this difference may have been the result of a general methodological phenomenon.

**Summary:**

Based on studies of how survey questionnaires influence responses, it appears reasonable to claim that surveys are more likely to find exaggerated positive views. Conversely, there are some indications in the literature that focus groups may result in complaints and overly negative attitudes, but this is still an open question. We suggest that while problematic issues tend to be under-communicated in questionnaire surveys, they may be overstated in focus groups.

We argue for the importance of increasing our understanding of focus group methodology, for example by reporting interesting discrepancies in mixed methods studies. In addition, more experimental research on focus groups should be conducted to advance the methodology and to test our hypothesis.

## Background

After several years of conducting qualitative research as well as mixed methods research, combining broad surveys with in depth focus group studies, we have started reflecting upon an apparent tendency for focus groups to convey a more negative view of the topics in question than the views conveyed through surveys. Revisiting the material from our earlier focus group studies, we note that some participants have had similar thoughts when they have been asked to assess the focus group experience. Below is an extract from a focus group discussion among general practitioners where they comment on their own presentation of their relationship with hospital psychiatrists (study described in [1]):

"*K: Well, we are painting a rather gloomy picture here.*"

"*G: I think that we are kind of coming up with all the bad stuff. There are a lot of people who manage fine.*"

"*Several participants: Mmmm. Yes.*"

"*K: And not everyone we meet in the hospital sector is that difficult.*"

"*G: Yes, some of them are really nice.*"

These kinds of statements, combined with a comparison of survey results, have made us speculate: Could the focus group as a method be biased towards negative findings?

Quantitative and qualitative methods have complementary strengths and weaknesses, and researchers are therefore increasingly encouraged to mix these different approaches [2-6]. The manner in which (qualitative) focus group studies can enhance the validity and value of (quantitative) surveys has been described with particular enthusiasm [7,8]. Focus groups allow the researcher to get a more complex and complete picture of a phenomenon and is seen as a useful basis when developing valid questionnaires for surveys [9]. Surveys, on the other hand, can serve to map the distribution of findings from focus group studies.

Despite growing support for mixed methods approaches we still have little systematic knowledge about the consequences of combining research methods in general, and focus groups and surveys specifically [10]. Because of a lack of empirical research on focus group methodology, we know far less about the mechanisms that characterise focus group discussions than we know about respondents’ reactions to questionnaire surveys. Psychology and the social sciences have a long tradition of survey methodology research [11-13], and already in the early 1970s, Sudman and Bradburn [14] were able to include more than 800 sources in their review of studies of survey response effects. However, with a few exceptions [15-17], no such interest has been given to the advancement of focus group methodology [10,18,19]. Some researchers have described how focus group studies utilize small group dynamics to extract other types of and additional information to surveys, but authors disagree about the kind of information this involves and how to best combine methods [3]. As Morgan and others have repeatedly pointed out, assertions about how focus groups work best and their limitations are usually based on intuition and personal experience rather than empirical evidence [3,10,15,18-20].

As a basis for our discussion, we briefly present an example from our own research where our use of both focus groups and a questionnaire survey led to results that appeared to be pointing in different directions and where the focus group findings conveyed a much more negative view than the survey. The findings, which were reported in separate publications [21-24], appeared plausible and were supported by other studies on the same topic and using the same methodology. In addition, important issues that were raised in the focus group study seemed to be confirmed as valid by the survey. It was not until we compared the main messages from our two studies that we were struck by the discrepancy, and even conflict between them. With reference to existing research on survey respondent behaviour and small group dynamics we discuss these differences; and we suggest how more research on focus group methodology is needed to further our understanding.

### Methods and results of the mixed methods study

In our first study we conducted focus groups with Norwegian general practitioners (GPs). The aim of the focus group study was to elicit GPs’ attitudes to clinical guidelines and guideline adherence. In the second study, we conducted a survey where key postulations were directly drawn from the focus group discussions. The survey aimed to explore the importance and distribution of attitudes to guidelines that we had identified in the focus group study. Further details of both parts of the study have been reported elsewhere [21,22,24].

### The focus groups

We conducted six focus groups with a total of 27 GPs in 2007. Our sampling strategy was a mixture of convenience and purposive sampling. We mailed a general invitation to participate in the study to the leaders of 93 doctors' educational groups. A majority of Norwegian GPs participate in these groups during their careers, either to obtain a specialist certification in general practice or to maintain the specialist competence. Eleven educational groups responded to the invitation and we selected six of these groups with a total of 27 members. The six groups were chosen in order to achieve a sample that was fairly similar to the population of GPs with regard to age, gender, professional experience, list size, urbanisation, and patient populations of different socioeconomic levels. Interviews were carried out until no new themes occurred according to a continuous and preliminary analysis.

The participants in each group knew each other from regular group meetings. The focus groups were moderated by one of the authors (BC), who presented herself at the start of each group session as a social scientist funded by the Research Council of Norway, and informed participants about the motivation of the study and anonymity issues. The interview guide specified some overarching themes, including participants’ confidence in and adherence to clinical guidelines, and how such guidelines influence professional autonomy and shared decision making. The moderator asked the participants initially to define what they meant by guidelines, encouraged participants to discuss freely, and only probed to clarify and to secure that all the predefined themes were discussed. The moderator aimed at conveying a neutral stance towards the role of clinical guidelines in general practice. Participants were asked specifically for both positive and negative views and examples, but the moderator consistently began by asking participants to discuss what they perceived to be positive aspects of guidelines.

We applied thematic content analysis [25] to identify common themes and arguments. Two researchers read the transcripts from the discussions thoroughly and then discussed emergent themes and possible codes until agreement was reached.

A total of 27 GPs, 18 men and 9 women with a mean age of 45 years, participated in the focus groups. In comparison, the mean age in the national population of GPs is 47. The sex ratio and size of patient lists (work load) were similar to those of the study sample [26].

We registered lively discussions in the groups; the moderator did not dominate the discussions and talked less than any single participant. The GPs referred primarily to a limited number of well-known guidelines, including guidelines for primary prevention of cardiovascular disease, diabetes, antenatal care, COPD/asthma and mammography screening. In line with earlier international studies, several barriers to following guidelines emerged, including a lack of trust in the evidence behind the guidelines, a desire to adjust treatment to the needs of the individual patient, and different practical challenges. The most striking and consistent message focus group participants gave was one of general scepticism towards guidelines and their authors. The doctors were particularly suspicious of health authorities’ possible economic motives behind their guidelines, and this was presented as a reason for non-adherence.

Our main impression from the focus group interviews was that negative views dominated, and participants’ negative statements were both longer and more emotional than their positive remarks. This impression was based on our observations during the interviews, on further interpretations while listening to the recordings of the interviews and on our analyses of the transcriptions. In addition, we coded and quantified what we perceived to be positive and negative remarks about guidelines, and counted 69 positive and 46 negative remarks. While the strength of participants’ scepticism is difficult to convey, the statement below, where a doctor describes his relationship to the health authorities, captures some of the atmosphere:

"*Dr N: To me, it’s essentially about feeling part of a team. I don’t feel we are part of a team, we are opponents. We each have our own team and we are doing the best we can, at least I am. I’m not so sure about them. But I don’t feel they are very interested in working with me or with us as a group. That’s very negative of me, but it’s how I feel.*"

"(Carlsen 2010:264)"

### The survey

The survey was carried out a year later, in 2008. Using the key findings from the focus group study, we developed a questionnaire to explore how the attitudes we had identified were distributed among Norwegian GPs. We asked about adherence to and confidence in clinical guidelines in general, presenting a broad definition of guidelines, and did not mention any particular guideline as an example. We also asked about confidence in guideline authors, such as the health authorities. Key postulations that the respondents were asked about their agreement with were:

·I have good knowledge of guidelines in my specialty

·Generally, I follow the guidelines

·I have confidence in guidelines from the health authorities

·I have confidence in guidelines from the Medical Association

·There are too many guidelines in my specialty

·The distinction between information, guidelines and regulations is unclear

·Guidelines are frequently difficult to access

We also asked the respondents to rate the importance of the following obstacles when guidelines are ignored:

·I lack a comprehensive, definitive source of guidelines

·There are several competing guidelines in my specialty

·Inconsistent guidelines are confusing

·The guideline does not fit the individual patient

·Guidelines are only suggestions, clinical judgement should be applied

·Economic concerns overshadow clinical concerns

·I am sceptical about the evidence

·The recommendation is contrary to the patient’s preferences

We distributed the questionnaire to a representative sample of 1600 Norwegian medical doctors, including 400 GPs. The questionnaire was part of a more extensive panel survey administered by The Research Institute of the Norwegian Medical Association every other year. The response rate was 60 % among the GPs in the survey. The respondents were informed about anonymity issues when they originally entered the panel. We also had access to anonymous background data about the respondents and their practices.

The survey results showed that almost all of the respondents (98 %) claimed that they generally followed guidelines, while 88 % said that they had confidence in guidelines issued by the health authorities. Hence, the survey, in contrast to the focus group study, indicated that almost all the GPs were generally positive to clinical guidelines issued by the authorities. However, the barriers to guideline adherence that emerged in the focus group study were confirmed to be of importance and the survey also confirmed the relative importance of the different barriers. Thus, the barriers identified in the focus group study were confirmed as relevant by survey participants, but appeared to have little impact on participants’ confidence in the guidelines and the health authorities.

## Discussion

### How can the differences be explained?

We interpret the main finding in our comparison above as an indication that the attitudes of the samples are comparable, and that the difference is not one of essence, but rather one of degree. This difference between the focus groups and the survey could reflect methodological flaws tied to these two particular studies such as the representativeness of the small and self-selected focus group sample. More detailed discussions of the strengths and limitations of the two studies can be found in the corresponding publications [22,24], but a few key problems need to be mentioned here:

The survey is based on a representative sample with an acceptable response rate, and the sample in the focus group study is quite similar to the sample in the survey according to some observable variables. Thus both survey and focus group participants share specific background characteristics with the general GP population. While the survey respondents may be more motivated then the non-respondents, this motivation is not necessarily tied to the questions about guidelines as this only constituted a small part of the panel study. The focus group participants, on the other hand, may be seen as less representative than the survey participants because of the lower number of participants and stronger issues of self-selection. Although the focus group participants were similar to the survey participants in terms of certain observable background characteristics, we know less about their characteristics in terms of motivation, personalities and general attitudes, and it could be argued that such a self-selected sample is often made up of individuals with a *message*. However, in this case, we invited the groups via the group leaders, who are responsible for setting up a programme for each group session of the educational group. We therefore expect that the group participants may have had varying levels of interest in our endeavour. As for the group leaders, they may have had especially strong opinions about the subject but could also simply have been motivated by the offer to be spared the work of planning a group session. Moreover, an advantage of focus groups is the opportunity to ask participants to explain their motivations. Our initial questions regarding motivation led us to believe that the group leaders were more motivated by a general interest in improving practice than in guidelines in particular.

We have also considered how the researchers’ may have influenced the results of the focus group study. When scrutinising the transcripts again in light of the survey results we could not see that the researcher tended to ask leading questions or probe for negative views. Instead, in our opinion, the researcher held a rather low profile during the group discussions. However, due to the fact that the researchers enjoy more flexibility when interpreting qualitative material than they do when interpreting quantitative data, emotional utterings or detailed stories from a few convincing informants may influence the researchers to draw too strong or even distorted conclusions. While we recognise our limited ability to assess our own research, we did take the recommended precautions in the analysis (e.g. recording the discussions and involving another researcher who had not participated in data collection). Also, in the re-analysis done for these methodological reflections, we introduced an external researcher (CG), who had neither been involved in data collection or analysis of the two primary studies.

It is also worth noting that although the survey depicts more positive attitudes towards guidelines, the barriers identified in the focus groups are strongly supported in the survey, and the relative importance of the barriers is the same in the two studies. In addition, the findings in both studies are supported by other studies from other researchers.

We therefore suggest that both studies are basically methodologically sound and that the discrepancy in results therefore is not primarily caused by contextual methodological limitations.

An alternative explanation for these differences could be that they are an indication of a general methodological phenomenon: that the two data collection methods tend to bring forward different attitudes. Below we refer to empirical research which suggests that problematic issues are under-communicated in surveys. Our experiences from mixing these two methods suggest that focus groups, in contrast, appear to overstate problematic issues. Does existing literature on survey and focus group methodology support this hypothesis?

### Do surveys paint too bright a picture?

As mentioned above, the literature indicates that survey respondents tend to understate problems and negative feelings [11]. Survey participants’ desire to please researchers in surveys has been documented in a range of studies under different headings such as *courtesy bias*[2,27] and *socially desirable responses*[28]. Researchers using survey methods have sometimes made use of observational data to compare people’s actual behaviour with the behaviour they have described in the survey. With regard to our own research theme, various studies have documented that survey respondents predominantly answer positively to general questions about guideline adherence [29], and exaggerate their own guideline adherence rates [30].

It may be argued that these general characteristics, or biases, of surveys alone could explain the discrepancy in our results. However, considering the apparently striking difference in the findings of our studies and the fact that we have very little research-based knowledge about the typical workings of focus groups, we believe it is possible that the focus group method has its own biases.

### Do focus groups paint too gloomy a picture?

It is of course possible that focus groups also generate courtesy bias, i.e. that participants understate problems and over-report socially desirable behaviour. This has been discussed as a hypothetical problem [20], but empirical verification is lacking. Conversely, it has been argued that the fact that the researcher(s) is outnumbered in focus groups leads to a conformity bias amongst the participants rather than an urge to please the researcher(s) [20,31,32], but, again, there is a lack of empirical data to support this hypothesis.

Faced with a lack of direct evidence with which to better understand focus group processes specifically, some researchers have turned to the field of small group dynamics [20]. Within this field, it has been demonstrated that we have a tendency to hide differences of opinion when we participate in group sessions [33], a phenomenon that is incorporated in the term g*roup conformity.* As focus groups are supposed to be well suited to capture the common norms of a social group [31,34], group conformity may in fact be employed as an important tool in focus groups [35]. Researchers are commonly advised to place homogenous participants in the same focus group, which could reinforce such tendencies [36]. Denzin and Lincoln suggest that in focus groups, “collective identities are consolidated” (p904). Robson claims that focus groups lead to consensus and that deviant views tend to be “weeded out” [37](p284). In much the same way, Albrecht argues that focus group participants “discuss an issue and offer a unified voice in presenting their opinions to the researcher” [20] (p53). In contrast, Morgan and Krueger argue that focus groups are not comparable to natural groups in that they are monitored and that, according to their experiences, the tendency to conformity in focus groups is merely a myth [38]. This view is partly supported by one of the few methodological reports from a focus group study, which offers examples of how “brave individuals” challenge the dominant view of the group [17]. But still, data is lacking as focus group discussions have not been subjected to empirical research to the extent that other group processes have.

Another interesting finding in empirical research regarding group behaviour is the phenomenon that social groups reinforce attitudinal tendencies and thus lead the group towards extreme views [39] or *group polarization*[10,40]. Hogg and Turner [41] argue that group polarization is the effect of conformity to a norm that defines the participants’ own group in contrast to other groups. A related theme is *exaggeration bias*[42] where group dynamics lead to a “bandwagon effect”, with group members endorsing more extreme ideas than they would express individually [34]. This view is supported by more recent research where it is noted that group participation may lead to a ”movement to more extreme positions” [43] (p46).

With regard to focus groups, some authors [44] have argued that the presence of other participants in the group may prevent participants from exaggerating. However, inspired by small group research, Sussman et al. [40] compared focus group discussions with participants’ responses in a survey before and after the focus groups and found that participants acquired more extreme attitudes after discussing the relevant subject in a focus group. The authors note that there seems to be an amplification of the prevalent group norms, or a “polarization effect”.

But could these group conformity and polarization effects take the form of a negative attitude bias in focus group studies? After carrying out a focus group study of school students’ views of their science classes, Watts and Ebbutt [45] suggested that focus groups encourage more critical comments than for example individual interviews: “Many people, and our youngsters may be no exception, once set upon a critical path, enjoy the opportunity of a collective 'moan session” (p32). The authors then go on to describe these group discussions as an “infectious downward spiral of shaded awfulness.” Watts and Ebbut’s paper is widely referred to, and several authors have used their assertion to explain an excess of negative comments in their focus groups. However, we have found no study involving empirical comparisons between methods that could support this claim.

A related mechanism could be that focus groups may be experienced as particularly well suited for forwarding complaints. Participants are offered time and encouragement to explain their attitudes and personal experiences regarding a problem area in detail to external researchers who, in addition, may be connected to the relevant authorities/policy makers, as we were in this focus group study. This could make the focus group a good opportunity for those who want to forward a message of complaint about a difficult situation or an unjust practice. It is therefore essential that focus group researchers report and reflect upon how participants are recruited, what motivated them to participate and that the researchers discuss and reflect upon who they represent towards the participants. In our particular comparison, we have taken account of these issues and, as mentioned, found no strong reason to suspect that the participants were driven by a wish to complain to start out with. We suggest instead that the participants’ complaints may be a characteristic of the group process rather than a reflection of their original motivation to participate in the focus groups.

### Methodological considerations

It has been argued that the potential of mixed methods is not realised in practice, e.g. that data from randomised controlled trials are seldom integrated with attached qualitative data [46]. Could this be due to difficulties in comparing findings from different types of studies? This question is related to the basic debate about whether different methods offer completely different types of data, which therefore are difficult to compare, or whether they instead can yield comparable and thus, either supportive or conflicting data. Many qualitative researchers would argue in favour of the first postulation; that the questions asked and the findings drawn from focus group studies and surveys are of different natures and thus incomparable. If we consider the epistemology of the first postulation to be a relativist stance, and the second a positivist stance, we would argue for the pragmatic middle position, of *subtle realism*[47]. We acknowledge that one research study will convey one of multiple existing perspectives, but argue that all perspectives are not equally important. While there is no eternally true, perfect story, some stories are less partial, less distorted and more *relevant* than others [48]. One consequence of this view of science is that we attempt to select the methods most appropriate to the research aim. In this study, our aim was to find out whether the views expressed by focus group participants were typical of the general GP population. Here, the decision to compare focus group and survey data was a direct result of the research question and on how we planned to use the data. The questions in the survey were designed as postulations drawn directly from the summarised postulations that came up in the focus groups as we found that it was relevant and possible to check the distribution of these attitudes through the survey. Hence, the findings in the two studies are of the same type, and *comparable* in that respect. A different question is whether one would expect findings stemming from a small convenience sample to be reproduced in a broad, representative sample. In this case, the details of the barriers to guideline adherence were to a large extent confirmed in the survey, whereas the main impression or finding; the scepticism of governmental guidelines, was not confirmed. Adding this to other anecdotal observations from our work with focus groups, we found it reasonable to at least pose the question of a possible general phenomenon in focus groups.

Focus group methodology is an understudied area and our hypothesis rests mainly on only one empirical study and on inferences from research into small group dynamics. We are aware that knowledge about other types of small groups is not necessarily valid for focus groups. While extensive research into survey methodology suggests that surveys lead to overly positive findings, it is possible that our hypothesis regarding focus groups is false and that this method is not particularly liable to either overly positive or overly negative data. We have suggested alternative explanations; e.g. systematic tendencies regarding motivation among participants or researchers in FG studies, but still our hypothesis is plausible and may form part of the hitherto unexplored methodological characteristics of FGs.

### Conclusive remarks and way forward

In our original presentation of our own survey findings [22], we made the reservation that people generally respond more positively in surveys than observations of their actions indicate. However, when presenting the focus group data, we did not discuss the possibility that the focus groups could have given an overly negative impression. Hereafter, we may want to consider making such reservations in focus group studies.

Methodological studies of questionnaire surveys have shed light on respondent behaviour; knowledge which is actively used by researchers when designing surveys and interpreting results. Methodological studies of focus groups could help researchers in similar ways, and could possibly facilitate integration of findings from qualitative and quantitative parts of mixed methods studies. Methodological studies of focus groups could include, for example, experimental studies that explore how focus groups influence participants’ attitudes; studies where actual behaviour and behaviour as reported in focus groups is compared; or, as Hyde et al. (2005) suggest, studies where individual participants are asked in post-session questionnaires to validate the group discussion. In addition, we encourage researchers of mixed method studies to explore whether there are systematic differences between findings produced by the survey and the focus group study that may be related to distinctive characteristics of the methods involved.

## Summary

We conducted a focus group study and a follow up questionnaire survey on the same topic, doctors’ attitudes to clinical guidelines, but the two parts of the study indicated conflicting conclusions. In contrast to the survey, the focus group study indicated that doctors were generally negative to clinical guidelines. We had reason to believe that both studies were methodologically sound. Combined with our experience from several focus group studies, this led us to speculate whether the focus group setting encourages participants to exaggerate views in a negative direction. We discuss the possibility that while problematic issues tend to be under-communicated in questionnaire surveys, they may be overstated in focus group interviews. While surveys have been the subject of much empirical research, we know less about focus group processes. Based on the methodological literature, it is reasonable to claim that surveys, especially when based on general questions, tend to find positive views. There are some indications in the literature that focus groups may result in elaborated complaints, but this is still an open question. We argue for the importance of increasing our understanding of focus group methodology, for example by reporting interesting discrepancies in mixed methods studies. Also, more experimental research on focus groups should be conducted to advance the methodology and test our hypothesis.

## Competing interests

Both authors declare that they have no competing interests.

## Authors' contributions

BC initiated the paper. Both authors discussed and developed the idea. BC drafted the paper and both authors have contributed to writing the paper. Both authors read and approved the final manuscript.

## Authors' information

Both authors are social anthropologists with PhDs within health services research from the medical faculties of the Universities of Bergen and Oslo. Both authors have experience in the use and interpretation of individual interviews and focus groups and have published mainly in health related journals. Carlsen has also experience in the use of surveys. The authors have also taught and published work within research methodology, including mixed methods and quantitative and qualitative meta-studies.

## Pre-publication history

The pre-publication history for this paper can be accessed here:

http://www.biomedcentral.com/1471-2288/12/134/prepub
